# Cancer stem cell-derived CHI3L1 activates the MAF/CTLA4 signaling pathway to promote immune escape in triple-negative breast cancer

**DOI:** 10.1186/s12967-023-04532-6

**Published:** 2023-10-14

**Authors:** Shufeng Ji, Hao Yu, Dan Zhou, Xulong Fan, Yan Duan, Yijiang Tan, Min Lang, Guoli Shao

**Affiliations:** 1 Special Medical Service Center, General Surgery, Zhujiang Hospital of Southern Medical University, No. 253, Middle Gongye Avenue, Haizhu District, Guangzhou, 510280 Guangdong People’s Republic of China; 2 Department of Breast Surgery, The First People’s Hospital of Foshan, Foshan, 528000 People’s Republic of China; 3 Department of Breast Surgery, Maternity and Children’s Healthcare Hospital of Foshan, Foshan, 528000 People’s Republic of China

**Keywords:** CHI3L1, CTLA4, MAF, S100A4, Triple-negative breast cancer, Cancer stem cells, T cells, Immune escape

## Abstract

**Background:**

Triple-negative breast cancer (TNBC) development may be associated with tumor immune escape. This study explores whether the CHI3L1/MAF/CTLA4/S100A4 axis affects immune escape in TNBC through interplay with triple-negative breast cancer stem cells (TN-BCSCs).

**Objective:**

The aim of this study is to utilize single-cell transcriptome sequencing (scRNA-seq) to uncover the molecular mechanisms by which the CHI3L1/MAF/CTLA4 signaling pathway may mediate immune evasion in triple-negative breast cancer through the interaction between tumor stem cells (CSCs) and immune cells.

**Methods:**

Cell subsets in TNBC tissues were obtained through scRNA-seq, followed by screening differentially expressed genes in TN-BCSCs and B.C.s (CD44^+^ and CD24^−^) and predicting the transcription factor regulated by CHI3L1. Effect of CHI3L1 on the stemness phenotype of TNBC cells investigated. Effects of BCSCs-231-derived CHI3L1 on CTLA4 expression in T cells were explored after co-culture of BCSCs-231 cells obtained from microsphere culture of TN-BCSCs with T cells. BCSCs-231-treated T cells were co-cultured with CD8^+^ T cells to explore the resultant effect on T cell cytotoxicity. An orthotopic B.C. transplanted tumor model in mice with humanized immune systems was constructed, in which the Role of CHI3L1/MAF/CTLA4 in the immune escape of TNBC was explored.

**Results:**

Eight cell subsets were found in the TNBC tissues, and the existence of TN-BCSCs was observed in the epithelial cell subset. CHI3L1 was related to the stemness phenotype of TNBC cells. TN-BCSC-derived CHI3L1 increased CTLA4 expression in T cells through MAF, inhibiting CD8^+^ T cell cytotoxicity and inducing immunosuppression. Furthermore, the CTLA4^+^ T cells might secrete S100A4 to promote the stemness phenotype of TNBC cells.

**Conclusions:**

TN-BCSC-derived CHI3L1 upregulates CTLA4 expression in T cells through MAF, suppressing the function of CD8^+^ T cells, which promotes the immune escape of TNBC.

**Supplementary Information:**

The online version contains supplementary material available at 10.1186/s12967-023-04532-6.

## Introduction

Triple-negative breast cancer (TNBC) is a subtype of breast cancer in which the expression of E.R. (estrogen receptor), P.R. (progesterone receptor), and HER2 (human epidermal growth factor receptor 2) is negative [1, 2]. Triple-negative breast cancer stem cells (TN-BCSCs) exert a critical function in the initiation, therapy resistance, and progression of TNBC [3]. Immune escape is a crucial process in the malignant development of tumors and poses a significant obstacle to immunotherapy [4]. Immune escape can enable a B.C. cell to form recurrence or metastasis [5]. Therefore, it is essential to further the understanding of the molecular mechanism of immune escape in TNBC.

The cancer stem-like cells (CSCs) possess high self-renewal ability and tumor-initiating capability, playing a crucial role in tumor immune suppression and contributing to tumor recurrence, metastasis, amplification, drug resistance, and relapse [6, 7]. Triple-negative breast cancer (TNBC) is prone to metastasis and recurrence [8]. Recent research has revealed that tumor stem cells in triple-negative breast cancer exhibit increased therapeutic tolerance and metastatic ability, thereby facilitating TNBC metastasis and recurrence [9]. CTLA-4 is primarily expressed on activated T cells and can restrict the interaction between CD28 and CD80/CD86 expressed on antigen-presenting cells (APCs) surface through competitive binding, thereby leading to immune suppression [10]. Numerous studies have shown that immune checkpoint blockade of CTLA-4 can unleash T cell-mediated therapeutic responses against cancer [11]. Furthermore, the Role of CTLA-4 and its immune checkpoint in breast cancer treatment has also demonstrated potential [12]. Currently, the mechanisms underlying the effect of CTLA-4 in triple-negative breast cancer and its interaction with TNBC tumor stem cells remain unclear. Exploring these underlying mechanisms could be valuable in identifying therapeutic targets for triple-negative breast cancer.

This study aimed to investigate the potential molecular mechanisms underlying the immune escape of triple-negative breast cancer (TNBC) through the interaction between tumor stem cell-like cells and immune cells, mediated by the CHI3L1/MAF/CTLA4 signaling pathway, using single-cell transcriptomic sequencing technology. Using annotation and clustering methods, various cell subtypes in TNBC tissue were identified, and TN-BCSCs were further selected from the CD44 + & CD24- cell subpopulation. The final results indicated that CHI3L1 derived from TN-BCSCs upregulated CTLA4 expression in T cells through MAF, leading to CD8 + T cell suppression and facilitating TNBC immune evasion. Additionally, this research provides new targets and strategies for treating TNBC and holds significance for investigating the immune escape mechanisms of various tumors, including breast cancer.

## Materials and methods

### Ethical approval

The study was carried out with the approval of the Ethics Committee of Zhujiang Hospital at Southern Medical University. The procedures *for the Care and Use of Laboratory Animals*, as outlined in the Guide, were followed during the animal experiment. Every effort was made to minimize the suffering experienced by the experimental animals.

### scRNA-seq data acquisition and cell grouping

The scRNA-seq dataset GSE180286 [13] was obtained from the GEO database. Two primary TNBC samples, GSM5457199 (P1) and GSM5457208 (P4), were analyzed using the “Seurat” package in the R software [14]. Quality control measures were applied to the data, requiring nFeature_RNA > 50, nCount_RNA < 20000, and percent.mt < 20. Genes exhibiting highly variable expression (top 2000 variance) were selected, and a linear reduction was performed using principal component analysis (PCA). The resulting PCA was then used to cluster PC_1-PC_20 with the tSNE algorithm. Cell types were annotated using the “SingleR” package in the R software, with “ref_Human_all” as the reference dataset [15].

### Screening and analysis of the differentially expressed genes (DEGs)

Differentially Expressed Genes (DEGs) were identified using the “limma” package in the R software [16]. DEGs were screened in two comparisons: between TN-BCSCs and B.C.s with the criteria of |logFC|> 0.3 and FDR < 0.05, and between CTLA4 + T and CTLA4- T with the criteria of |logFC|> 0.5 and FDR < 0.05. For visual representation, the “pheatmap” package was employed. Additionally, the “clusterProfiler” package was used for performing Gene Ontology (G.O.) enrichment analysis. The "VennDiagram" package was utilized to identify genes that intersect between different sets. This package enabled the creation of Venn diagrams to visualize the overlapping genes.

### Cell culture and transfection

Human triple-negative breast cancer cell lines, MDA-MB-231 (ATCC, HTB-26, USA) and MDA-MB-453 (ATCC, HTB-131, USA), were cultivated using DMEM-H (Thermo Fisher, 10100147C, USA) medium supplemented with 10% FBS (Thermo Fisher, 11965092, USA). Human spleen-derived T cells (MingZhou Bio, 4229, Zhejiang, China) along with human CD8^+^ T cells were cultured using CTS™ OpTmizer™ T cell expansion SFM medium (Thermo Fisher, A1048501, USA). Isolation of human CD8^+^ T cells from T cells was performed using the Dynabeads™ CD8 Positive Isolation Kit (Thermo Fisher, 113333D, USA). All cell cultures were supplemented with 1% Penicillin–Streptomycin (Thermo Fisher, 15140163, USA), and the cells were maintained in an incubator at 37 °C with a 5% CO2 atmosphere.

A lentivirus-mediated infection protocol was used to construct the following cell lines: CHI3L1-overexpressed MDA-MB-231 and MDA-MB-453 cell lines (or-CHI3L1-231 and oe-CHI3L1-453) and their negative control (N.C.) cell lines (oe-NC-231 and oe-NC-453), MAF-knockdown T cell lines (sh-MAF-1 T and sh-MAF-2 T) and their N.C. cell lines (sh-NC T). Sangon (Shanghai, China) provided plasmid construction and lentivirus packaging, and shRNA sequences are detailed in Additional file 8: Table S1. The constructed plasmids (oe-NC, oe-CHI3L1, sh-NC, sh-MAF-1, and sh-MAF-2) were co-transfected into 293T cells, and packaged lentiviruses were obtained after verification, amplification and purification. For lentiviral infection, 5 × 10^5^ cells were seeded into 6-well plates. When cell confluency reached 70–90%, the cells were infected with a medium containing an appropriately packaged lentivirus (MOI = 10; working titer: approximately 5 × 10^6^ TU/mL) and 5 μg/mL polybrene (TR-1003, Merck KGaA, Darmstadt, Germany). After 48 h of infection,1 μg/mL puromycin (A1113803, Thermo Fisher Scientific) was used to obtain stably infected cell lines. As described above, the fluorescence-labeled cells (MDA-MB-231-EGFP and MDA-MB-453-EGFP) used for the animal experiments were also constructed.

### Spheroid formation assay

Cells were inoculated into 6-well plates pre-packaged with Pol y-HEMA (Merck, P3932, USA) in an inoculum quantity of 5 × 10^3^ cells/well. Cells were treated with B-27 (1:50, Thermo Fisher, A1895601, USA), 0.4% BSA (Merck, A1933, USA), 20 ng/ml EGF (Merck, SRP3027, USA), 20 ng/ml basic-FGF (PeproTech, 100-18B, USA), 5 μg/ml insulin (Merck, I3536, USA), and 1% Penicillin–Streptomycin in DMEM/F-12 (Thermo Fisher, A4192001, USA) in culture for approximately 8 days to allow the production of mammary microspheres.

### CCK-8 and colony formation assays

CK-8 kit (Biyuntian, C0037, Shanghai, China) was used to detect cell proliferation. Logarithmic growth phase cells were taken and adjusted to a concentration of 5 × 10^4^ cells/mL with DMEM-H medium containing 10% FBS, then spread into a 96-well culture plate, 100 μL of cell suspension was added to each well and incubated in an incubator for 48 h. After the supernatant was quickly discarded, 10 μL of CCK-8 solution was added to each well and incubated at 37 °C for 2 h. The absorbance values at 450 nm (A) were measured using a Multiskan FC enzyme marker ( Thermo Fisher, 51119080, USA) to determine the absorbance value at 450 nm (A). Three parallel wells were set up in each group, and the average values were taken.

Cells in the logarithmic growth phase were detached and subcultured to make cell suspension. Next, 5 mL cell suspension was seeded into the plate with a density of 100 cells/plate. After incubation at 37 °C with 5% CO_2_ for 2–3 weeks, methanol was used for fixation for 15 min, followed by staining with Giemsa solution for 10 min. The number of colonies with greater than 10 cells was counted by the naked eye or under a microscope, and the colony formation rate was calculated.

### Scratch assay to detect cell migration

Previous studies [17] were referenced to conduct the scratch assay experiment. The procedure is briefly described: horizontal lines were marked on the back of a 6-well plate using a marker pen, with 1 cm spacing between each line. Each line was ensured to pass through the corresponding sample well. At least 4 lines were marked within each sample well. Approximately 5 × 10^5^ cells were evenly distributed within each well and allowed to adhere for 12 h. Wells showing 100% cell confluence were selected for subsequent experiments. After incubation for 24 h, a white pipette tip with a 10 μL capacity was used to vertically scratch the lines marked on the back of the wells. The scratch area was washed with PBS to remove detached cells, repeated three times, and then replenished with a serum-free culture medium. The scratched 6-well plate was placed in a 37 °C, 5% CO_2_ incubator for 24 h, and samples were taken for cell imaging. Cell images were analyzed using ImageJ software to calculate the average distance between cells.

### Transwell assays for cell migration and invasion

Logarithmic growth phase cells were taken, digested and washed once with PBS and serum-free medium successively. The cells were suspended with serum-free medium, counted, and the concentration was adjusted to 2 × 10^5^ /mL. For the invasion assay, Matrigel gel (Corning, 356,237, USA) was removed from − 20 °C in the refrigerator at 4 °C overnight. Matrigel gel was diluted to 300 μL/mL with serum-free cell culture medium at 4 °C, and 100 μL was evenly applied to a layer of The cell culture cell was then gently placed into the wells of the 24-well plate and placed at 37 °C for about 3 h. The cell culture cell was taken out and dried on the ultra-clean bench overnight, and the migration experiment was carried out directly in the next step. Add 800 μL of conventional medium containing 10% serum to the lower chamber (i.e., the bottom of the 24-well plate) and 150 μL of the cell suspension to the upper chamber and continue to incubate in the incubator for 24 h. Aspirate and discard the residual liquid from the upper and lower chambers, and gently wipe away the residual gel and uninvaded cells from the upper chamber with a clean cotton swab, paying attention to avoid damaging the basement membrane; wash the upper and lower chambers with PBS twice, and aspirate and discard the PBS; add 0.5 mL 4% paraformaldehyde, fixed for 30 min and then aspirated. Add 0.5 mL 0.1% crystalline violet, stain for 20 min and aspirate, wash 3 times with PBS, then microscopically examine and count the number of cells on the lower surface of PET membrane, count the middle and surrounding 5 fields of view and take the average.

### Cell treatment

First, 0.4 μm Transwell was used to co-culture tumor and T cells. T cells were seeded in the Transwell apical chamber (1 × 10^5^ cells/well), and MDA-MB-231 or BCSCs-231 cells were seeded in the Transwell basolateral chamber (5 × 10^4^ cells/well). In the CHI3L1 blocking experiment, 20 μg/mL anti-CHI3L1 (MAB25991, R&D Systems, Minneapolis, MN) was added to the basolateral chamber with BCSCs-231 cells (BCSCs-231 + anti-CHI3L1). To verify MAF's effect, sh-NC or sh-MAF-1 (T cells) were seeded in the Transwell apical chamber and BCSCs-231 in the basolateral chamber. Cells were harvested after 48 h for subsequent analysis.

To verify the immunosuppressive effect of CTLA4^+^ T cells, CD8^+^ T cells (10^4^ cells/96well) were treated with untreated T cells (Mock), and MDA-MB-231 or BCSCs-231 treated T cells (MDA-MB-231-T or BCSCs-231-T, 10^3^ cells/96well), respectively, in co-cultured in medium containing Dynabeads™ human T activator CD3/CD28 (Thermo Fisher, 11132D, USA) and hrIL-2 (20 I.U./mL, Thermo Fisher, PHC0021, USA). For exogenous treatments, 20 μg/mL anti-CTLA4 ( Bio X Cell, BN13, USA) (BCSCs-231-T + anti-CTLA4), 5 μg/mL hrCD86 (MCE, HY-P7323, USA) (BCSCs-231-T + hrCD86 and hrCD86) were added respectively The cells were collected after 48 h for Flow cytometric analysis to assess the cytotoxicity of CD8^+^ T cells.

For other cell treatments, 1 μg/mL hrCHI3L1 (MCE, HY-P70030, USA), 20 μg/mL anti-S100A4 (R&D, MAB4137, USA), and 2 μg/mL hrS100A4 (MCE, HY-P71140, USA) were added to the corresponding culture systems, respectively The cells were collected after 48 h of incubation for subsequent analysis.

### Flow cytometric analysis and apoptosis detection

Cell samples were washed in PBS and resuspended; tissue samples were digested in PBS containing 0.8 mg/mL Collagenase IV ( Merck, C4-BIOC, USA) for 30 min at 37 °C, the tissue supernatant was collected, centrifuged at 850 × *g* for 10 min, washed in PBS, and resuspended using Percoll (Merck, P1644, USA) to remove dead cells and then resuspended. The EGFP + tumor cells were sorted out using flow cytometry before detecting the proportion of TN-BCSCs in the tissue samples. When intracellular antigen detection was required, cells were permeabilized with 0.5% Tween 20 (Merck, P2287, USA) for 5 min before incubation with primary antibody. Samples were incubated with the antibody at 4 °C and analyzed using flow cytometry (B.D. Bioscience, BD LSRFortessa, USA). pe-anti-Human-CD44 (10 μL/106 cells, Abcam, ab269300, U.K.) and Alexa Fluor^®^ 647-Mouse-anti-Human-CD24 (20 μL/test, B.D. Bioscience, 561644, USA) were used to detect the proportion of TN-BCSCs in the samples; APC- Mouse-anti-Human-CTLA4 (20 μL/test, B.D. Bioscience, 555855, USA) and FITC-anti-Human-CD3 (1.0 μg/test, Abcam, ab34275, U.K.) were used to detect the proportion of surface CTLA4^+^ T cells in the samples; FITC -Mouse-anti-Human-Granzyme B (GzmB) (20 μL/test, B.D. Bioscience, 560211, USA) or Alexa Fluor® 488-Mouse-anti-Human-Ki67 (5 μL/test, B.D. Bioscience 561165, USA) and APC-Mouse-anti-Human-CD8 (5 μL/test, B.D. Bioscience, 340584, USA) was used to assess the cytotoxicity of CD8^+^ T. The obtained data were analyzed using BD FACSDiva software.

Flow-through apoptosis assays were performed using the Pacific Blue™ Membrane Linker V/SYTOX™ AADvanced™ Apoptosis Kit (Thermo, A35136, USA), following the protocol provided by the manufacturer.

### ELISA

Cell culture supernatants (conditioned medium, CM) were collected, and samples were assayed for CHI3L1 (Thermo Fisher, EHCHI3L1X5, USA) and S100A4 (Abcam, ab283547, U.K.) expression levels using enzyme-linked immunosorbent assay (ELISA) kits according to the manufacturer’s instructions.) expression levels.

### Western blot

Total proteins were extracted from the cells and tissues using the Protein Extraction Kit (BB3101, Bestbio, Shanghai, China), and protein concentrations were quantified by BCA Protein Assay (P0012S, Beyotime). Proteins were loaded on SDS-PAGE gels for transfer to a PVDF membrane, followed by blockade with TBST containing 5% skim milk powder and incubation with the primary antibodies (Additional file 8: Table S2) and then with HRP-labeled goat anti-rabbit IgG (1:2000, ab6721, Abcam) or HRP-labeled goat anti-mouse IgG (1:2000, ab6789, Abcam). β-actin served as the internal reference. ECL reaction solution (P0018FS, Beyotime) was used for color development.

### RNA extraction and RT-qPCR

Total RNA was extracted using Trizol (16096020, Thermo Fisher Scientific) following the manufacturer's guidelines. Next, the RNA was reversely transcribed into cDNA using the R.T. reagent Kit (RR047A, Takara, Shiga, Japan). Gene expression analysis was performed using SYBR® Premix Ex TaqTM II kits (DRR081, Takara) on the real-time fluorescence qPCR instrument (ABI 7500, Thermo Fisher Scientific). With β-actin as the internal reference, gene expression was quantified by the 2^−ΔΔCt^ method. The list of primers is provided in Additional file 8: Table S3.

### Breast cancer in situ transplantation model construction and treatment

Forty-eight 4–5-week-old immune system humanized female mice weighing 20 ± 2 g were purchased from Beijing Viton Lever Laboratory Animal Technology Co., Ltd (408, huPBMC-NOG, Beijing, China). Mice were housed in standard feeding cages at a constant room temperature of 23 ± 1 °C with 12–12 h of light-darkness and free access to food and water. The mice were acclimatized for one week before the experiment. Our hospital animal ethics committee approved this experimental procedure and animal use protocol.

Twenty-four mice were randomly selected and divided into 3 groups: MDA-MB-231, BCSCs-231 and BCSCs-231 + anti-CHI3L1, and inoculated with MDA-MB-231-EGFP or BCSCs-231-EGFP (obtained by enrichment in MDA-MB-231-EGFP) in the mammary fat pad of mice, respectively. Amount 5 × 10^5^. BCSCs-231 + anti-CHI3L1 group was injected with anti-CHI3L1 (0.5 mg/kg) via the tail vein twice weekly. Tumour growth was assessed weekly using a bioluminescence imaging system (PerkinElmer, IVIS Imaging System Xenogen, USA) and plotted growth curves. Mice were euthanized 35 days (5 weeks) after tumor inoculation, and tissues were taken to detect relevant indicators.

The remaining 24 mice were randomly divided into 3 groups: Mock + sh-NC, hrCHI3L1 + sh-NC, and hrCHI3L1 + sh-MAF-1, and inoculated with MDA-MB-453-EGFP in the mammary fat pad in a quantity of 5 × 10^5^. Packaged adenovirus of sh-NC or sh-MAF-1 was injected 48 h after injection (AVV-6. 10^11^ UT, 50 μL, provided by Bioengineering) once every 2 weeks. Tail vein injection of hrCHI3L1 (0.1 mg/kg MCE, HY-P70030, USA) was given 48 h after the first injection of sh-NC or sh-MAF-1 twice a week. Mice were euthanized 35 days (5 weeks) after tumor inoculation, and tissues were taken to detect relevant indexes.

### Statistical analysis

All statistical analyses of the data in this study were performed using SPSS 26.0 (IBM, USA) statistical software. The measurement data were expressed as mean ± standard deviation. Normality and chi-square tests were first performed for conformity to a normal distribution and chi-square, unpaired t-tests were used between groups. One-way ANOVA or ANOVA of repeated measures data was used for comparison between multiple groups. p < 0.05 indicated that the differences were statistically significant.

## Results

### CHI3L1 involved in immune evasion of BCSCs

Cancer Stem Cells (CSCs) exhibit remarkable self-renewal capabilities and possess the capacity to initiate tumorigenesis, thus holding a pivotal role in fostering tumor immunosuppression [6]. While monoclonal antibodies targeting CTLA4 have found applications in treating cancers like kidney and lung malignancies [18, 19], the connection between Triple-Negative Breast Cancer Stem Cells (TN-BCSCs) and CTLA4 remains inadequately elucidated.

To identify the target genes that regulate CTLA4 in TN-BCSCs, we initially performed tumor tissue clustering in TNBC. To investigate whether TN-BCSCs can participate in tumor immune escape via CTLA4, we obtained the breast cancer-associated single-cell RNA sequencing (scRNA-seq) dataset GSE180286 from Gene Expression Omnibus (GEO). We analyzed two primary TNBC samples, GSM5457199 (P1) and GSM5457208 (P4). We first looked at the number of genes (nFeature_RNA), the number of mRNA molecules (nCount_RNA) and the percentage of mitochondrial genes for all cells in the scRNA-seq data, and the results showed that most cells with nFeature_RNA < 6000, nCount_RNA < 20,000, and percent.mt < 25% (Additional file 1: Fig. S1A). We filtered the data with nFeature_RNA > 50 & nCount_RNA < 20,000 & percent.mt < 20 as criteria, and finally obtained the expression matrix of 6676 cells and 20,854 genes. The sequencing depth correlation calculation results showed the correlation coefficient r = 0.18 between nCount_RNA and percent. Mt for the filtered data and r = 0.92 between nCount_RNA and nFeature_RNA (Additional file 1: Fig. S1B) indicated that the filtered cell data were of good quality and could be used in the subsequent analysis.

**Fig. 1:**
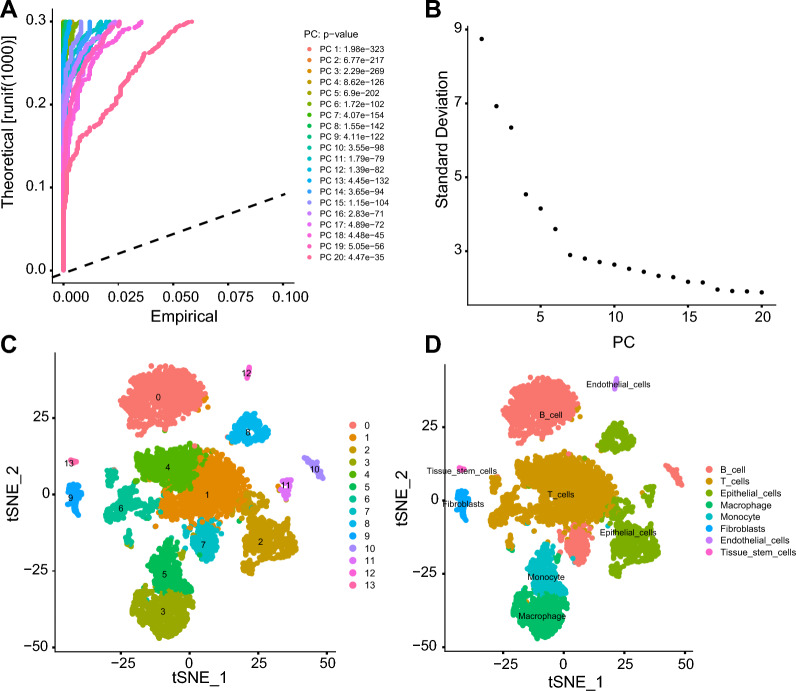
Cell clustering and annotation of the TNBC scRNA-seq data. **A** The position of the *p*-value of PC_1 and PC_20 in the PCA relative to the uniform distribution (dashed line). Significant Pcs are located above the dashed line, with a smaller *p*-value. **B** distribution of the standard deviation of the P.C.s. Significant P.C.s have a larger standard deviation. **C** Visualization of the tSNE clustering results, with a two-dimensional display of aggregation and distribution of the cells, in which each color represents a cluster. **D** Visualization of the cell annotation results, in which each color represents a cell subset

The gene expression variability in the data was calculated based on the gene expression variance data, and the genes with the top 2000 variance were selected as highly variable genes (Additional file 1: Fig. S1C). The central genes associated with PC_1-PC_4 obtained through linear dimension reduction and their expression heatmaps are shown in Additional file 1: Fig. S1D, E, and the distribution of cells in PC_1 and PC_2 is revealed in Additional file 1: Fig. S1F. The *p*-value distribution of P.C. _1 PC_20 was visualized using the JackStrawPlot function to obtain the position of P.C.s relative to the uniform distribution (dashed line) (Fig. 1A). The standard deviation of P.C.s was sorted using ElbowPlot (Fig. 1B). The results show that PC_1—PC_20 can fully reflect the information contained in the selected highly variable genes and have good analytical significance (Fig. 1A, B).

A total of 14 clusters (Fig. 1C) were obtained by clustering after dimension reduction of the top 20 P.C.s, and the gene expression heatmap of these 14 clusters was plotted (Additional file 2: Fig. S2). Subsequently, we used the R software “SingleR” package to annotate the cell types with "ref_Human_all" as a reference [15], and 8 cell subsets were obtained (Fig. 1D), including B cells, T cells, epithelial cells, macrophages, monocytes, fibroblasts, endothelial cells, and tissue stem cells. Specifically, clusters 0, 7 and 10 were B cells; clusters 1, 4 and 6 were T cells; clusters 2, 8 and 11 were epithelial cells; cluster 3 indicated macrophages; cluster 5 indicated monocytes; cluster 9 indicated fibroblasts; cluster 12 indicated endothelial cells; cluster 13 indicated tissue stem cells.

The above results indicate that triple-negative breast cancer tumor tissue can be divided into 14 clusters containing 8 cell subpopulations.

### The CTLA4-related gene CHI3L1 was highly expressed in TN-BCSCs

**Fig. 2:**
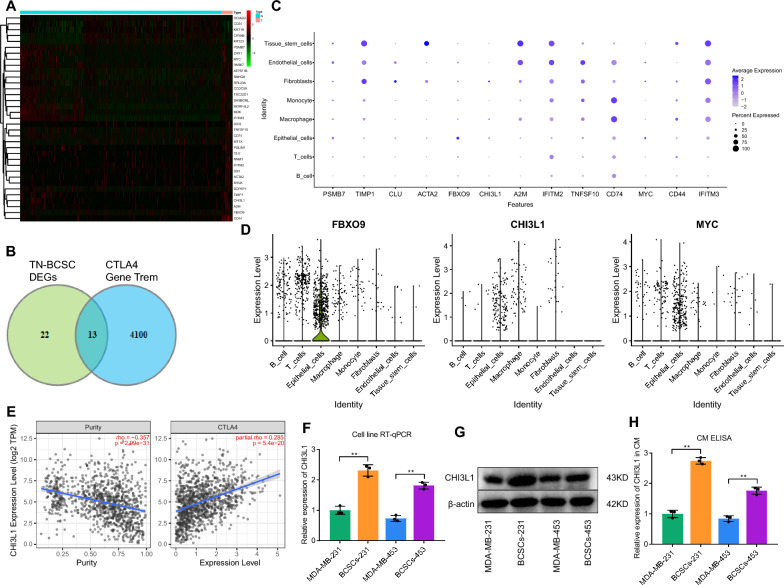
CTLA4-related gene expression in TN-BCSCs. **A** Expression heatmap of the DEGs between TN-BCSCs and B.C.s (CD44^−^ and CD24^+^). The red indicates the upregulated expression, and the green indicates the downregulated expression. **B** Intersection between DEGs in TN-BCSC and CTLA4-related genes (CTLA4 Gene Term). **C** Expression distribution plot of CTLA4-related DEGs in TN-BCSCs in eight cell subsets. **D** Violin plots of FBXO9, CHI3L1 and MYC. **E** The correlation between CHI3L1 and CTLA4 expression in B.C. was obtained from the TIMER2.0 database. The result is corrected by tumor purity. **F** and **G** The CHI3L1 expression in BCSCs obtained from MDA-MB-231 and MDA-MB-453 cells and the parental cells of BCSCs as measured by RT-qPCR and Western blot. **H** The expression of CHI3L1 in the supernatant of conditioned medium of BCSCs obtained from MDA-MB-231 and MDA-MB-453 cells and the parental cells of BCSCs as determined by ELISA. ^ns^ *p* > 0.05, * *p* < 0.05, ** *p* < 0.01, *** *p* < 0.001. Cell experiments were repeated three times

To screen TN-BCSCs from epithelial cell subsets, we obtained the human BCSC marker gene sets from CellMarker [20]. Comparing the expression of the top 10 marker genes (CD44, CD24, ALDH1, CD133, SOX2, ESA, EpCAM, OCT4, ALDH and ALDH1A1) in the scRNA-seq data, we found that CD44 and CD24 were expressed in the Epithelial_cells subpopulation. ALDH1A1 was not expressed in the Epithelial_cells subpopulation, while no other genes were detected (Additional file 3: Fig. S3A, B). CD44^+^/CD24^−^ phenotype is reliable for screening BCSCs [21], and thus based on the median values of CD44 + and CD24- expression, TN-BCSCs (CD44^+^ and CD24^−^) were sorted from the epithelial_cells subset (Additional file 3: Fig. S3C). Subsequently, differential analysis was performed using CD44^−^ & CD24^+^ cells (i.e., B.C.s) in the Epithelial_cells subpopulation as control samples, and 35 differentially expressed genes (DEGs) were obtained (containing 30 upregulated genes and 5 down-regulated genes) (Fig. 2A, Additional file 3: Fig. S3D). G.O. enrichment analysis of the DEGs (Additional file 3: Fig. S3E, F) showed that compared to BCs, TN-BCSCs were enriched in biological processes such as regulation of intrinsic apoptotic signaling pathway by p53 class mediator, response to interferon-gamma, and regulation of viral entry into the host cell. Meanwhile, there was also a lack of genes associated with response to estrogen, intermediate filament organization and intermediate filament cytoskeleton organization.

CTLA-4 is primarily expressed in T cells, and there is a low expression of CTLA-4 in TNBC and TN-BCSC [10]. Our single-cell sequencing analysis further supports this finding (Additional file 5: Fig. S5A). Further, we obtained CTLA4 Gene Term from GeneCards search, which contains 4113 CTLA4-related genes. Taking the intersection of TN-BCSC DEGs and CTLA4 Gene Term, we obtained 13 CTLA4-related candidate genes differentially expressed in TN-BCSCs (PSMB7, TIMP1, CLU, ACTA2, FBXO9, CHI3L1, A2M, IFITM2, TNFSF10, CD74, MYC (CD44 and IFITM3) (Fig. 2B). We analyzed the expression of these 13 genes in 8 cell subpopulations and the results showed that FBXO9, CHI3L1 and MYC were mainly expressed in Epithelial_cells with reasonable specificity (Fig. 2C, D, Additional file 3: Fig. S3G). Subsequently, we obtained the expression correlation of these three genes with CTLA4 in breast cancer online from TIMER2.0 [22]. The results showed that CHI3L1 and MYC were significantly and positively correlated with CTLA4 expression, with CHI3L1 being the most correlated with CTLA4 (rho = 0.285, p < 0.001) and no significant correlation between FBXO9 and CTLA4 (Fig. 2E, Additional file 3: Fig. S3H, I).

To verify the expression of CHI3L1 in TN-BCSCs, we enriched BCSCs from TNBC cell lines MDA-MB-231 and MDA-MB-453 using microsphere culture. Identification results showed that the enriched BCSCs were microsphere-like in culture with phenotype CD44^+^ & CD24^−^, consistent with the characteristics of BCSCs (Additional file 3: Fig. S3J-L). Subsequently, we examined the expression of CHI3L1 in BCSCs with their parental cells. RT-qPCR and Western blot showed that CHI3L1 expression was significantly higher in TN-BCSCs obtained from MDA-MB-231 and MDA-MB-453 enrichment than in parental cells (Fig. 2F, G). ELISA results showed that The level of CHI3L1 in the supernatant of TN-BCSCs medium (conditioned medium, CM) was significantly higher than that in the CM of their parental cells (Fig. 2H).

Collectively, the CTLA4-related gene CHI3L1 was upregulated in TN-BCSCs.

### CHI3L1 promotes TNBC stemness

**Fig. 3:**
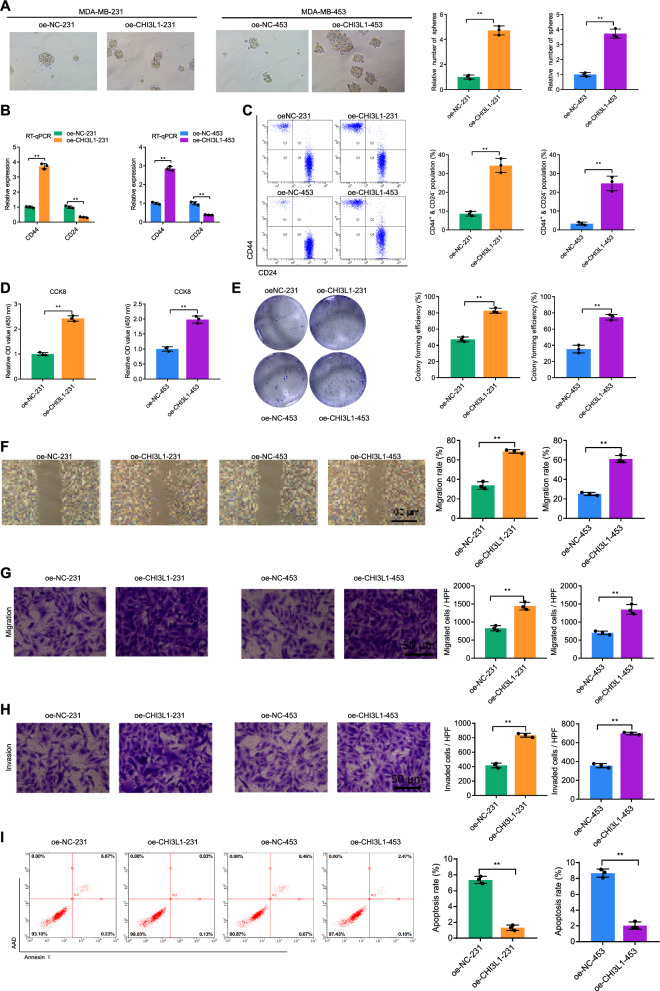
Effects of CHI3L1 overexpression on the stemness phenotype of TNBC cells. **A** Representative results of spheroid formation assay to detect the stemness phenotype of MDA-MB-231 and MDA-MB-453 cells after CHI3L1 overexpression (left) and the quantitative chart (right). **B** RT-qPCR to detect BCSC markers CD44 and CD24 expression in MDA-MB-231 and MDA-MB-453 cells after CHI3L1 overexpression. **C** Flow cytometry to measure the proportion of BCSCs (CD44^+^ and CD24^−^) in MDA-MB-231 and MDA-MB-453 cells after CHI3L1 overexpression. **D** and **E**, Colony formation assay and CCK-8 assay to detect MDA-MB-231 and MDA-MB-453 cell proliferation after CHI3L1 overexpression. **F**–**H** Scratch assay and Transwell assay to detect MDA-MB-231 and MDA-MB-453 cell migration and invasion abilities after CHI3L1 overexpression. **I** Flow cytometry to measure MDA-MB-231 and MDA-MB-453 cell apoptosis after CHI3L1 overexpression. ^ns^ *p* > 0.05, * *p* < 0.05, ** *p* < 0.01, *** *p* < 0.001. Cell experiments were repeated three times

Compared with normal breast cells (B.C.s), breast cancer stem cells (BCSCs) exhibit increased proliferation, migration, invasion, and resistance to apoptosis [23]. Several studies have shown that CHI3L1 can promote the stem-like properties of ovarian cancer cells, leading to poor prognosis [24]. To investigate whether CHI3L1 can promote TNBC stemness, we established CHI3L1 overexpressing cell lines based on MDA-MB-231 and MDA-MB-453 cell lines. The successful construction of these cell lines was confirmed by RT-qPCR, Western blot, and ELISA (Additional file 4: Fig. S4A–C). Additionally, sphere formation assay and flow cytometry results demonstrated that CHI3L1 overexpression increased TN-BCSCs (Fig. 3A–C). To further validate the promotive effect of CHI3L1 on TNBC stemness, we performed the following experiments. CCK-8 and colony formation assays revealed a significant increase in proliferation and colony formation ability in oe-CHI3L1-231 and oe-CHI3L1-453 cells compared to oe-NC-231 and oe-NC-453 cells (Fig. 3D, E). Scratch and Transwell assays showed that overexpression of CHI3L1 significantly enhanced the migration and invasion abilities of MDA-MB-231 and MDA-MB-453 cells (Fig. 3E–H). Moreover, flow cytometry analysis demonstrated a lower proportion of apoptotic cells in the oe-CHI3L1 group compared to the oe-NC group, indicating that CHI3L1 overexpression could significantly increase the anti-apoptotic ability of MDA-MB-231 and MDA-MB-453 cells ( Fig. 3I).

Taken together, these results indicate that CHI3L1 can significantly enhance the stemness of TNBC, characterized by an increase in the proportion of CD44 +  and CD24- cells, as well as increased proliferation, migration, invasion, and resistance to apoptosis.

### TN-BCSCs upregulated CTLA4 expression in T cells via the CHI3L1/MAF axis.

**Fig. 4:**
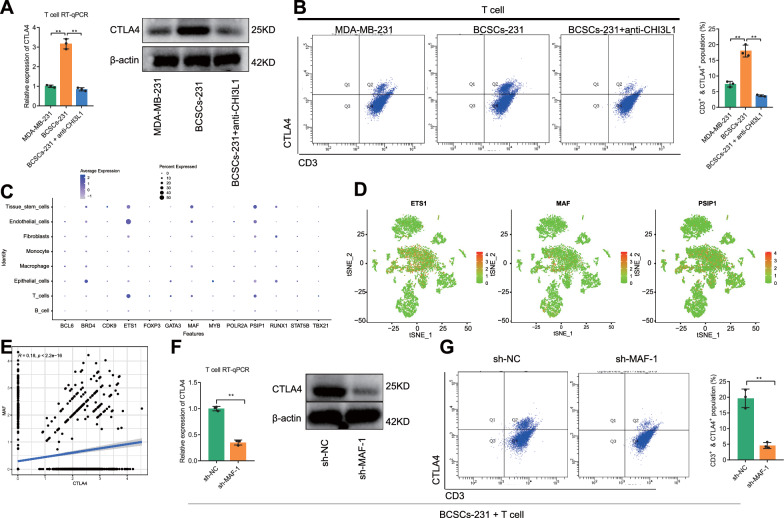
Effects of TN-BCSCs on CTLA4 expression in T cells. **A** RT-qPCR and Western blot to measure CTLA4 expression in T cells after co-culture with MDA-MB-231 or BCSCs-231 cells. **B** Representative results of CTLA4^+^ (on the cell surface) T cell proportion changes after co-culture (left) and quantitative chart (right). **C** Scatter plot showing the expression distribution of T cell-related transcription factors of CTLA4 predicted through the Cistrome D.B. database in cell subsets. **D** distribution of the predicted transcription factors of CTLA4 (ETS1, MAF and PSIP1) in the scRNA-seq data. **E** Correlation between MAF and CTLA4 expression in the T cell subset of the scRNA-seq data. **F** RT-qPCR and Western blot to detect CTLA4 expression change after co-culture of T cells with MAF knockdown and BCSCs-231 cells. **G** Representative results (left) and quantitative statistical chart (right) of the change in the proportion of CTLA4^+^ T cells after co-culture of T cells with MAF knockdown and BCSCs-231 as determined by flow cytometry. ^ns^ *p* > 0.05, * *p* < 0.05, ** *p* < 0.01, *** *p* < 0.001. Cell experiments were repeated three times

CTLA4 is mainly expressed in T cells [10], as demonstrated by the cited scRNA-seq data (Additional file 5: Fig. S5A). Before verifying whether TN-BCSCs can regulate CTLA4 expression in T cells via CHI3L1, we examined the original CTLA4^+^ T cell population in T cells using flow cytometry, and the results showed that only very few CTLA4 + T cells (CD3^+^ & CTLA4^+^) were present in the T cells we used (Additional file 5: Fig. S5B). The RT-qPCR and Western blot results showed that the expression of CTLA4 in T cells was significantly upregulated after co-culture with BCSCs-231 cells compared with that co-culture with MDA-MB-231 cells (Fig. 4A). Flow cytometric analysis showed that the percentage of surface CTLA4^+^ was increased in T cells co-cultured with BCSCs-231 (Fig. 4B). With anti-CHI3L1 added to the medium, CTLA4 expression was significantly inhibited (Fig. 4A, B), which indicated that TN-BCSCs might regulate CTLA4 expression in T cells mainly by secreting CHI3L1.

Further, we obtained the transcription factors predicted to regulate CTLA4 expression from the Cistrome D.B. database and later validated the expression of 15 of them associated with T Lymphocyte, Th1 and Th2 in the scRNA-seq data. The results showed that a total of 13 transcription factors were expressed in the scRNA-seq data (BCL6, BRD4, CDK9, ETS1, FOXP3, GATA3, MAF, MYB, POLR2A, PSIP1, RUNX1, STAT5B and TBX21), among which ETS1, MAF and PSIP1 were expressed in the T_cells subpopulation expression levels were higher in the T_cells subpopulation (Fig. 4C, D, Additional file 5: Fig. S5C). Using GTRD, the target genes of ETS1, MAF and PSIP1 were searched, and only MAF and CTLA4 were found to have a mutually regulatory relationship. Next, the JASPAR database was used to predict binding sites between MAF and the CTLA4 promoter region, and the potential binding sites with high scores were found (Additional file 5: Fig. S5D). Meanwhile, the correlation analysis showed a good correlation between MAF and CTLA4 in the T cell subset of TNBC scRNA-seq data (Fig. 4E, Additional file 5: Fig. S5E). Therefore, MAF in TNBC might be a key transcription factor regulating CTLA4 expression in T cells.

Based on the RT-qPCR and Western blot results, sh-MAF-1 and sh-MAF-2 had good knockdown efficiency, with sh-MAF-1 having optimal efficiency (Additional file 5: Fig. S5F, G). The results of RT-qPCR, Western blot and Flow cytometry showed that CTLA4 expression in T cells was significantly decreased by MAF-1 knockdown (Fig. 4F, G), indicating that TN-BCSCs might regulate CTLA4 expression in T cells through MAF.

To further validate the Role of CHI3L1 and reduce confounding factors, we repeated the above experiments using human recombinant CHI3L1 protein (hrCHI3L1) instead of BCSCs-231 and obtained the same results: CHI3L1 can upregulate CTLA4 expression in T cells, and the process may be mediated through MAF (Additional file 5: Fig. S5H–J).

The above results suggest that TN-BCSCs can secrete CHI3L1 to act on T cells and upregulate CTLA4 expression in T cells via MAF.

### TN-BCSCs suppress CD8 via CTLA4^+^ T cells^+^ T cell toxicity.

**Fig. 5:**
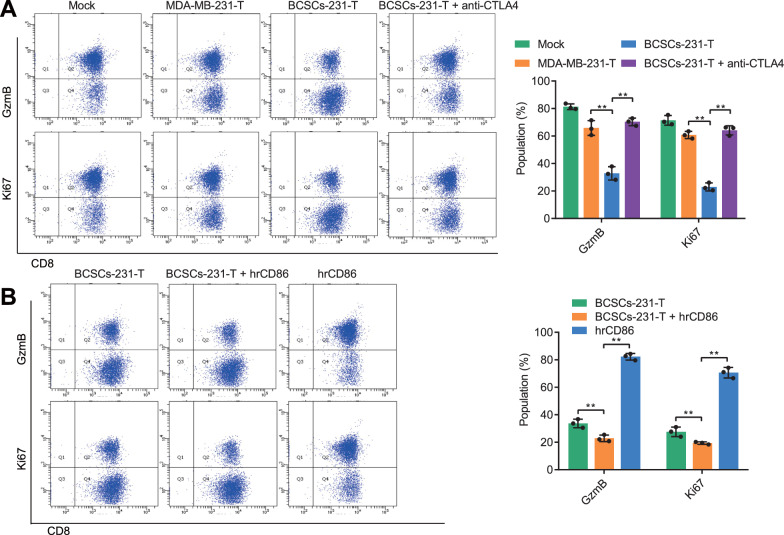
Effects of BCSCs-231-T on the function of CD8^+^ T cells. **A** Representative results (left) and quantitative statistical chart (right) of flow cytometry, showing the effect of BCSCs-231-T on GzmB and Ki67 expression in CD8^+^ T cells. **B** Representative results (left) and quantitative statistical chart (right) of the promoting Role of hrCD86 in CD8 + T cell dysfunction caused by BCSCs-231-T as detected by flow cytometry. ^ns^ *p* > 0.05, * *p* < 0.05, ** *p* < 0.01, *** *p* < 0.001. Cell experiments were repeated three times

We co-cultured T cell-treated BCSCs-231 cells (BCSCs-231-T cells) with CD8^+^ T cells to explore the Role of TN-BCSCs/CTLA4 axis in tumor immunosuppression. We found that compared with co-culture with MDA-MB-231-T cells, co-culture with BCSCs-231-T cells significantly inhibited the expression of cell cytotoxicity markers [25] GzmB and Ki67 in CD8^+^ T cells (Fig. 5A). When anti-CTLA4 was added exogenously, the inhibitory effect of BSCSs-231-T cells on CD8^+^ T cells was notably reduced (Fig. 5A), indicating that the immunosuppression due to TN-BCSCs was mainly mediated by CTLA4^+^ T cells.

CD80 and CD86 are ligands for CTLA4 and are expressed as markers of M2-type macrophages in various cancers [26]. In scRNA-seq data, CD86 was highly expressed in Macrophage and Monocyte cell subpopulations, while CD80 expression was relatively low (Additional file 6: Fig. S6A, B). Flow cytometry results further showed that the presence of CD86 in the co-culture system could significantly inhibit the expression of GzmB and Ki67 in CD8^+^ T cells. However, there was no significant inhibition when CD86 has added alone (Fig. 5B), indicating that CD86 acted via CTLA4.

The above results showed that CTLA4^+^ T cells generated by TN-BCSCs could inhibit the cytotoxicity of CD8^+^ T cells, and CD86 could enhance this inhibition.

### TN-BCSCs are involved in TNBC immune escape via CHI3L1/MAF.

To further verify that TN-BCSCs were involved in the immune escape of tumors via CHI3L1, we constructed an in situ transplantation model of breast cancer in mice with a humanized immune system using EGFP-labeled MDA-MB-231 and BCSCs-231. The experiments were divided into 3 groups: MDA-MB-231, BCSCs-231 and BCSCs-231 + anti-CHI3L1. Tumour growth was assessed weekly using small animal live imaging to detect bioluminescence intensity (BLI) at the transplantation site. Mice were euthanized 35 days after tumor inoculation, and tumor tissues were taken to detect relevant indexes. The results showed that the tumor growth rate and tumor size at the end of the experiment were significantly higher in the BCSCs-231 group than in the MDA-MB-231 group.

**Fig. 6:**
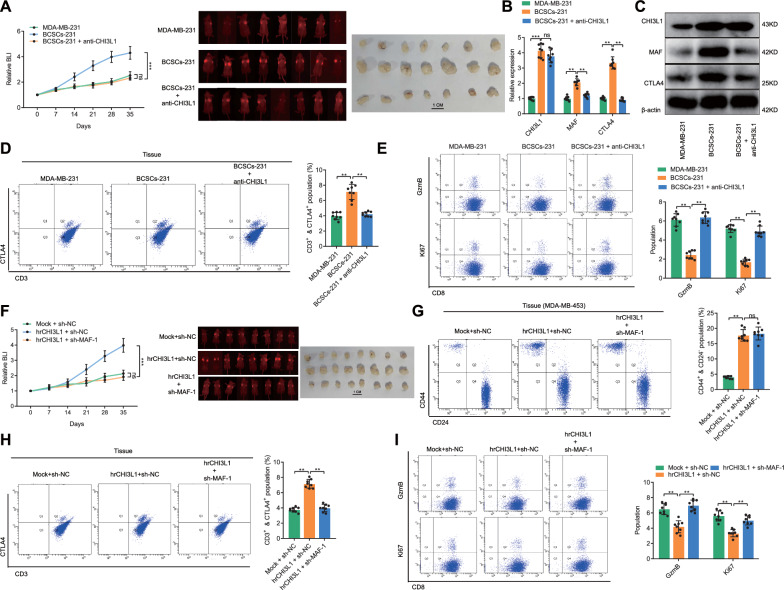
In vivo verification of the involvement of TN-BCSCs in immune escape of TNBC through CHI3L1/MAF. **A** Growth of MDA-MB-231 and BCSCs-231 orthotopic B.C. transplanted tumors. The tumor growth curve drawn based on the relative bioluminescence intensity is on the left. The bioluminescence intensity and tumor size at the end of the experiment are shown on the right side. **B** and **C** Detection of CHI3L1, MAF and CTLA4 expression in tumor tissues by RT-qPCR and Western blot. **D** and **E**, Detection of CTLA4^+^ T cell proportion and CD8^+^ T cytotoxicity (GzmB and Ki67) in tumor tissues by flow cytometry (n = 8). **F** Growth of MDA-MB-453 orthotopic B.C. transplanted tumor. The tumor growth curve drawn based on the relative bioluminescence intensity is on the left. The bioluminescence intensity and tumor size at the end of the experiment are shown on the right side (n = 8). **G** Flow cytometry to detect the proportion of BCSCs in MDA-MB-453 orthotopic B.C. transplanted tumors in mice treated with hrCHI3L1 or sh-MAF-1 (n = 8). **H** and **I**, Detection of CTLA4^+^ T proportion and CD8^+^ T cytotoxicity in MDA-MB-453 orthotopic B.C. transplanted tumor in mice treated with hrCHI3L1 or sh-MAF-1 by flow cytometry. ^ns^ *p* > 0.05, * *p* < 0.05, ** *p* < 0.01, *** *p* < 0.001

In comparison, the tumor growth rate and size in the BCSCs-231 + anti-CHI3L1 group were significantly lower than in the BCSCs-231 group (Fig. 6A). RT-qPCR and Western blot results showed that compared with the MDA-MB-231 group, CHI3L1, MAF and CTLA4 expressions were significantly upregulated in tumor tissues of BCSCs-231 group, and after exogenous administration of anti-CHI3L1, there was no significant change in CHI3L1 expression in BCSCs-231 + anti-CHI3L1 group compared with BCSCs-231 group, and MAF and CTLA4 expressions were significantly decreased (Fig. 6B, C). The results of Flow cytometric analysis showed that the proportion of CTLA4^+^ T cells in tumor tissues was significantly increased in the BCSCs-231 group compared with the MDA-MB-231 group, while the cytotoxicity of CD8^+^ T cells was suppressed; the proportion of CTLA4^+^ T cells in tumor tissues was significantly decreased in the BCSCs-231 + anti-CHI3L1 group compared with the BCSCs-231 group, while the cytotoxicity of CD8 T cells was suppressed in the BCSCs-231 group compared with the MDA-MB-231 group. Meanwhile, the cytotoxicity of CD8^+^ T cells was significantly increased (Fig. 6D, E).

Furthermore, we constructed a breast cancer in situ transplantation model using MDA-MB-453 (EGFP marker) to verify the Role of CHI3L1/MAF in promoting TNBC stemness and participating in immunosuppression by exogenously administering hrCHI3L1 or/and sh-MAF-1. Mice were randomly divided into 3 treatment groups: Mock + sh-NC, hrCHI3L1 + sh-NC and hrCHI3L1 + sh-MAF-1, and tumor growth was monitored using small animal live imaging. The results showed that the tumor growth rate and size in the hrCHI3L1 + sh-NC group were significantly higher than those in the Mock + sh-NC group. In comparison, the tumor growth rate and size in the hrCHI3L1 + sh-MAF-1 group were significantly lower than those in the hrCHI3L1 + sh-NC group (Fig. 6F). The mice were euthanized 35 days after tumor inoculation. Flow cytometric analysis later examined the proportion of BCSCs, the proportion of CTLA4^+^ T cells and the cytotoxicity of CD8^+^ T cells in the tumour tissues. The results showed that the proportion of BCSCs and the proportion of CTLA4 T cells in the tumor tissues of the^+^ hrCHI3L1 + sh-NC group were significantly increased, and the cytotoxicity of CD8^+^ T cells was reduced compared with that of the Mock + sh-NC group; after the exogenous administration of sh-MAF-1 to down-regulate the expression of MAF in the tumor tissues, the proportion of BCSCs in the hrCHI3L1 + sh-MAF-1 group was significantly increased compared with that of the hrCHI3L1 + sh-MAF-1 group. The ratio was not significantly different from that between the hrCHI3L1 + sh-NC group, while the proportion of CTLA4^+^ T cells was significantly reduced, and the cytotoxicity of CD8^+^ T cells was increased (Fig. 6G–I).

The above results suggested that TN-BCSCs promoted the expression of CTLA4 in T cells through CHI3L1/MAF axis, which inhibited the CD8^+^ T cell cytotoxicity, participating in immunosuppression in TNBC.

### TN-BCSCs-induced secretion of S100A4 by CTLA4^+^ T cells promoted the stemness phenotype of TNBC cells

**Fig. 7:**
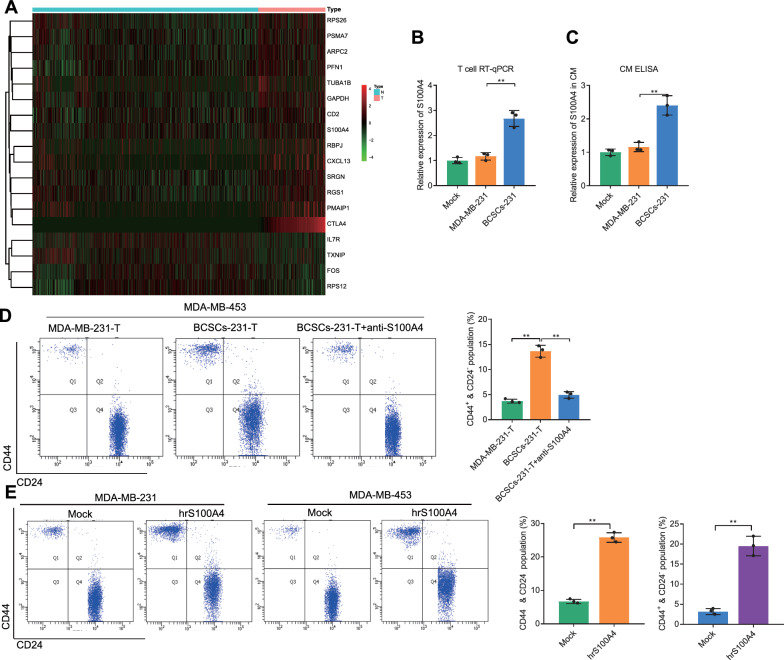
Effects of CTLA4^+^ T cells-secreted S100A4 on the stemness phenotype of TNBC cells. **A** Heatmap of DEGs in CTLA4^+^ T cells. Red represents upregulated genes, and green represents downregulated genes. **B** and **C** S100A4 expression change in T cells and the culture supernatants of T cells after co-culture with MDA-MB-231 or BCSCs-231 cells as detected by RT-qPCR and ELISA. **D** Flow cytometry to determine the effect of co-culture of MDA-MB-231-T or BCSCs-231-T with T cells on the proportion of CD44^+^ and CD24^−^ MDA-MB-453 cells. **E** Flow cytometry to determine the effect of exogenous hrS100A4 on the proportion of CD44^+^ and CD24^−^ MDA-MB-231 and MDA-MB-453 cells. ^ns^ *p* > 0.05, * *p* < 0.05, ** *p* < 0.01, *** *p* < 0.001. All cell experiments were repeated three times

To investigate the possibility that CTLA4^+^ T cells in TNBC promote tumor cell stemness. We divided the T_cells subpopulation into the CTLA4^+^ T group and CTLA4^−^ T group according to the presence or absence of CTLA4 expression. Subsequently, we performed differential gene expression analysis using CTLA4^−^ T as control and obtained 18 DEGs (containing 14 upregulated and 4 down-regulated genes) (Fig. 7A, Additional file 7: Fig. S7A). G.O. enrichment analysis of genes up- and down-regulated in CTLA4^+^ T cells, respectively, showed that compared to CTLA4^−^ T cells, CTLA4^+^ T cells were more efficient in myeloid dendritic cell activation, negative regulation of ossification and positive regulation of protein-containing complex assembly, and lack of response to progesterone, response to calcium ion and positive regulation of leukocyte differentiation and other functions (Additional file 7: Fig. S7B, C).

In the G.O. enrichment analysis of the CTLA4^+^ T cell upregulated gene, we found that S100A4 is associated with epithelial to mesenchymal transition, positive regulation of I-kappaB kinase/NF-kappaB signaling, and mesenchymal cell differentiation, which are tumor stem cell-associated biological processes, and there are also related studies suggesting that S100A4 is a potential factor in regulating tumor stem cells [27]. To verify whether TN-BCSCs-induced CTLA4^+^ T could promote TNBC cell stemness through S100A4, we first examined the changes in S100A4 expression in BCSCs-231-treated T cells (BCSCs-231-T). RT-qPCR and ELISA results showed that compared with the MDA-MB-231 group, BCSCs- 231 significantly upregulated the level of S100A4 in T cells and their culture medium supernatant (CM) (Fig. 7B, C). Subsequently, we co-cultured BCSCs-231-T with MDA-MB-453 and examined the changes in the expression of breast cancer stem cell markers in MDA-MB-453. The results of Flow cytometric analysis showed that the proportion of CD44^+^ & CD24^−^ cells in MDA-MB-453 was significantly higher in the BCSCs-231-T group than in the MDA-MB-231-T group, and exogenous addition of anti-S100A4 significantly inhibited this change (Fig. 7D). Moreover, in comparison with untreated T cells, hrS100A4 significantly upregulated the proportion of CD44^+^ and CD24^−^ MDA-MB-231 and MDA-MB-453 cells (Fig. 7E).

The above results suggested that TN-BCSCs-induced CTLA4^+^ T cells might promote the stemness phenotype of TNBC cells by secreting S100A4.

## Discussion

Immune escape plays a vital role in B.C., and the immune silence of B.C. is related to diminished immune recognition and promoted immunosuppression [28]. In this study, we used scRNA-seq to reveal the possible molecular mechanism of the CHI3L1/MAF/CTLA4/S100A4 axis in regulating the immune escape in TNBC. The results collectively demonstrated that this axis could promote immune escape in TNBC by mediating the interaction between TN-BCSCs and immune cells.

In the first place, we found in this study that TNBC tissues mainly contained 8 cell subsets. It was revealed that CHI3L1 was highly expressed in one of the cell subsets, TN-BCSCs and that CHI3L1 could promote the stemness of TNBC cells. Intriguingly, TN-BCSCs have been highlighted as essential regulators despite their capacity to induce the aggressiveness of TNBC [29]. Of note, CHI3L1 secreted by myeloid-derived suppressor cells was reported to augment the function of TN-BCSCs, which identified a nonimmunologic role for myeloid-derived suppressor cells in facilitating the progression of TNBC [30]. As previously reported, CHI3L1 could induce tumor immune escape in breast cancer by acting on the cytotoxic machinery while preventing granule polarization [31]. The induction of CHI3L1 expression could affect tumor-related immune responses and promote metastasis in breast cancer [32]. These studies support our results regarding the promoting Role of CHI3L1 in TNBC.

Furthermore, TN-BCSCs could secrete CHI3L1, which upregulated the expression of CTLA4 in T cells through MAF. CHI3L1 could be upregulated in the microenvironment dysfunction with PD-L1 expression, and blockade of PD-L1 could reverse CTLA4 immune checkpoint [33]. A high level of CTLA4 was found in CD69^+^ Tregs, and overexpression of CD69 could stimulate c-MAF expression [34]. Besides, increased c-MAF and CTLA4 expression co-existed in Treg-of-B cells [35]. The results of these previous reports suggest that there may be a possible interaction between CHI3L1 and CTLA4 and between CTLA4 and MAF, despite the absence of demonstration of the direct regulatory relationship between them.

It should be noted that the regulatory relationship between CHI3L1 and MAF has yet to be reported. It was revealed that MAFK, a MAF family member, was abundant in human TNBC and aggressive mouse mammary tumor cell lines. MAFK overexpression could induce epithelial-mesenchymal transition phenotypes of breast cancer cells while facilitating tumor formation [36]. Augmented c-MAF-inducing protein CMIP could negate the suppressed growth of TNBC induced by LINC01123 deficiency. Increased MAF oncogene expression was associated with the bone metastasis of BC [37]. Moreover, MAF amplification could diminish the treatment efficacy with adjuvant zoledronic acid in early BC [38]. Our study achieved the Role of CHI3L1 in TNBC through the MAF/CTLA4 axis.

We also demonstrated that TN-BCSCs promoted the expression of CTLA4 on T cells through CHI3L1/MAF axis, inhibited the CD8^+^ T cell cytotoxicity, and participated in TNBC immunosuppression. Immune escape is required for tumor progression, and current immune checkpoint therapy is based on enhancing CD8^+^ T cell cytotoxicity to eliminate cancer cells [39]. CTLA4-activated-like tumors manifested a conditional immune state resembling a cancer cell-exploited escape phenotype, and there existed meaningful interaction between tumor CLTA4-induced portraits and immune-infiltrating cells [40]. Interestingly, anti-CTLA4 combined with anti-PD-L1 could suppress tumor growth and increase survival in a TNBC model. This antitumor effect was achieved by recruiting CD8^+^ T and T memory cells to regulate the tumor microenvironment [41].

Moreover, anti-CTLA4 induced significant antitumor effects by promoting the infiltration of CD8^+^ T cells in TRAMP-C2-bearing C57BL/6j mice [42]. Increased cytotoxicity of CD8^+^ T cells was induced by simvastatin treatment, accompanied by downregulated CTLA4 expression [43]. Treatment of anti-CTLA4 in combination with cisplatin and anti-PD-1 enhanced antitumor immunity in TNBC, involved with activation of tumor-infiltrating cytotoxic CD8 and CD4 T cells [44]. The present study demonstrated that the CHI3L1/MAF/CTLA4 axis could inhibit CD8^+^ T cell cytotoxicity and induce TNBC immunosuppression.

Another critical finding obtained in this study was that TN-BCSCs-induced CTLA4^+^ T cells could secrete S100A4, which promoted the stemness phenotype of TNBC cells. S100A4 can appeal to T cells in primary and pre-metastatic tumors [45]. A previous study has shown exclusive expression of S100A4 by memory T cells of CD4^+^/CD8^+^ subsets [46]. Increased S100A4 expression was found in intraepithelial CD3-positive T lymphocytes [47]. Intriguingly, S100A4 was found to be directly bound by amlexanox, an azoxanthone drug that could exert anticancer functions through collaboration with anti-CTLA4 antibodies [48]. Prior research unfolded that activated S100A4 signaling due to PCDH7-regulated tumor-astrocyte interaction could retain the self-renewal of TN-BCSCs and modulate their adaptation and colonization [49]. Moreover, the S100A4 signaling was unveiled to enhance the invasive and intravasation capacities in geminin-overexpressing TNBC cells by promoting their stemness and epithelial-to-mesenchymal phenotypes [50]. However, these previous studies do not discuss the interaction between CTLA4 + T cells and S100A4 in the tumor microenvironment of TNBC.

**Fig. 8:**
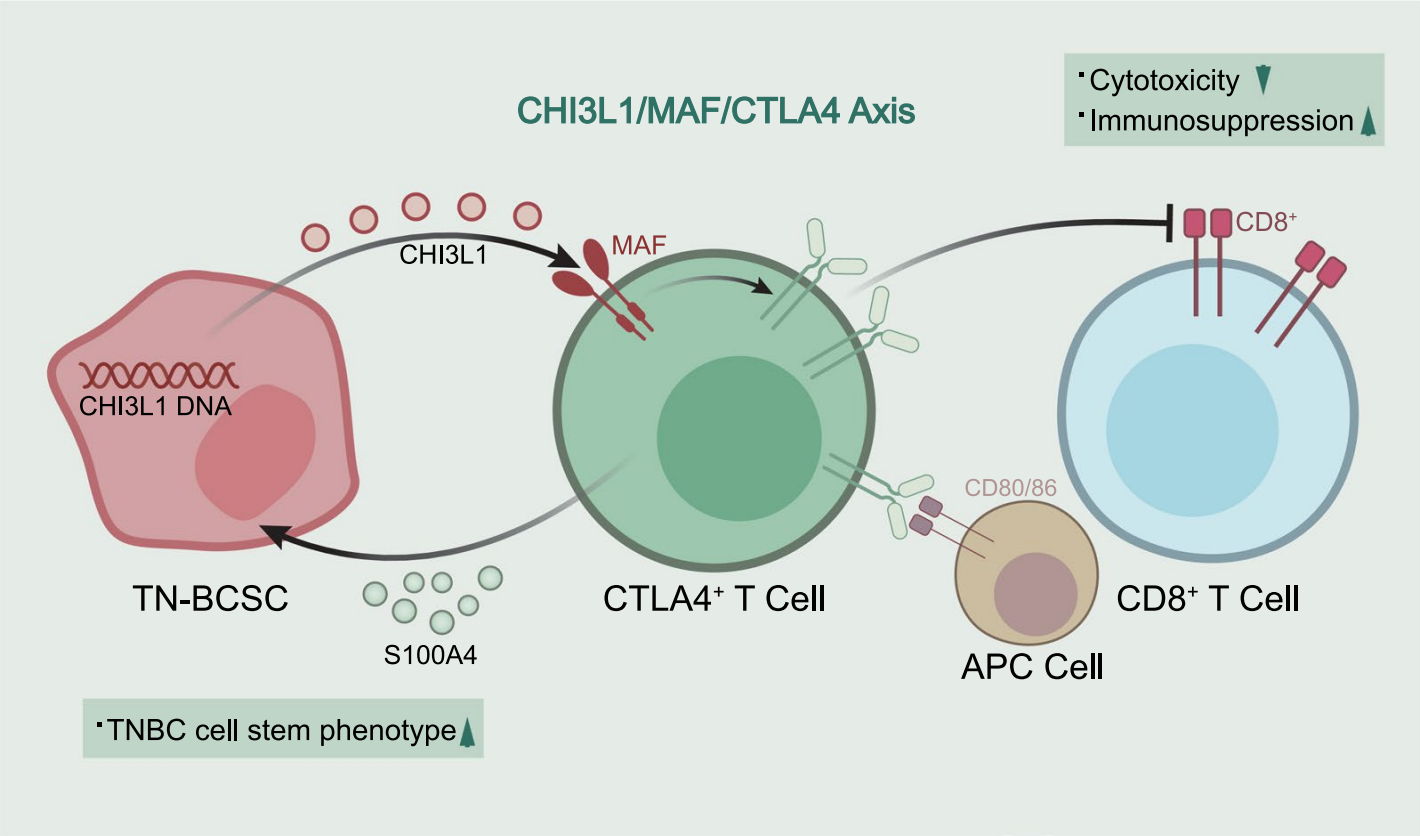
Schematic illustration of the molecular mechanism by which TN-BCSC-derived CHI3L1 promotes immune escape of TNBC via the MAF/CTLA4 axis. TNBC mainly contains 8 cell subsets, among which TN-BCSCs exist in the epithelial cell subgroup. As a characteristic gene expressed in TN-BCSCs, CHI3L1 is related to TNBC cell stemness. It upregulates the expression of CTLA4 in T cells through MAF, thereby inhibiting the cytotoxicity of CD8^+^ T cells and producing immunosuppression. CTLA4^+^ T cells promote the stemness phenotype of TNBC cells by secreting S100A4 through positive feedback

To conclude, TNBC mainly contained 8 cell subsets, among which TN-BCSCs existed in the epithelial cell subgroup. As a characteristic gene expressed in TN-BCSCs, CHI3L1 was related to TNBC cell stemness. It upregulated the expression of CTLA4 in T cells through MAF, thereby inhibiting CD8^+^ T cell cytotoxicity and inducing immunosuppression. CTLA4^+^ T cells promoted the stemness phenotype of TNBC cells by secreting S100A4 through positive feedback (Fig. 8). This study preliminarily revealed the molecular mechanism of BCSCs participating in TNBC immune escape through CHI3L1/MAF/CTLA4 axis and molecular target for basic research and clinical treatment of TNBC. However, the interaction between cells in the tumor microenvironment is quite complex, and this study only discusses a potential interaction mode between BCSCs and T cells. In addition, the specific molecular mechanisms of the interaction between CHI3L1 and MAF, the inhibition of CD8^+^ T cell function by CTLA4^+^ T cells, and the regulation of S100A4 on the stemness phenotype of TNBC cells remain elusive, which require further research.

### Supplementary Information

**Additional file 1: Figure S1.** TNBC scRNA-seq data quality control and PCA dimension reduction. A, Numbers of genes (nFeature_RNA) and mRNA molecules (nCount_RNA), and percentage of mitochondrial genes (percent. mt) per cell in the TNBC scRNA-seq data. B, Scatter plot of the correlation between nCount_RNA and percent.mt, nCount_RNA and nFeature_RNA in filtered data. C, ANOVA was performed to screen highly variable genes. Red represents the top 2000 highly variable genes, and black represents low variable genes. The gene names of the top 10 highly variable genes are marked. D, Top 30 related genes in PC_1 and PC_4 in PCA. E, Expression heatmap of top 30 related genes in PC_1 PC_4 in PCA. Yellow indicates the upregulated expression, and purple indicates the downregulated expression. F, distribution of cells in PC_1 and PC_2. Each dot represents one cell.

**Additional file 2: Figure S2.** Expression heatmap of top 10 DEGs in the 13 clusters obtained from tSNE clustering. Yellow color indicates upregulation, and purple color indicates downregulation.

**Additional file 3: Figure S3.** Expression of CTLA4-related genes in TN-BCSCs and enrichment identification of BCSCs. A & B, Scatter plots (A) and violin plots (B) showing the distribution of human breast BCSCs markers in cell subsets. C, Distribution of CD44 and CD24 expression in epithelial cell subsets. In the wireframe, TN-BCSCs (CD44^+^ and CD24^-^) have positive CD44 and CD24 expression at the median: D, Volcano plot of the expression of DEGs between TN-BCSCs and B.C.s. The red color represents the upregulated genes, and the green represents the downregulated genes. E & F, G.O. enrichment analysis results of upregulated genes (E) and downregulated genes (F) in the differential analysis between B.C.s and TN-BCSCs. G, Violin plot of the expression distribution of the CTLA4-related DEGs (PSMB7, TIMP1, CLU, ACTA2, A2M, IFITM2, TNFSF10, CD74, CD44, and IFITM3) in TN-BCSCs in each cell subset. H & I, The correlation of MYC with FBXO9 and CTLA4 expression in B.C. was analyzed through the TIMER2.0 database. The result is corrected by tumor purity. J, The morphological display of the BCSCs obtained from spheroid formation assay and their parental cells. K & L, Phenotypic identification of BCSCs obtained from spheroid formation assay and their parental cells. ^ns^ *p* > 0.05, * *p* < 0.05, ** *p* < 0.01, *** *p* < 0.001. Cell experiments were repeated three times.

**Additional file 4: Figure S4.** Validation of CHI3L1 overexpression in the TNBC cell lines. A & B, RT-qPCR (A) and Western blot (B) to verify the CHI3L1 overexpression in MDA-MB-231 and MDA-MB-453 cells. C, ELISA to detect CHI3L1 expression in the supernatant of the conditioned medium after CHI3L1 overexpression. ^ns^ *p* > 0.05, * *p* < 0.05, ** *p* < 0.01, *** *p* < 0.001. Cell experiments were repeated three times.

**Additional file 5: Figure S5.** Screening for the transcription factors of CTLA4 in T cells. A, Distribution of CTLA4 in scRNA-seq. B, Detection of the proportion of CTLA4^+^ untreated T cells by flow cytometry. C, Detection of the predicted transcription factors of CTLA4 in the scRNA-seq. D, MAF binding sequence identification map (left) obtained from the JASPAR website and predicted binding sites between MAF and CTLA4 promoter region (right). E, Correlation between ETS1, PSIP1 and CTLA4 expression in T cell subset in scRNA-seq. F & G, RT-qPCR and Western blot to verify the MAF knockdown efficiency in T cells. H & I, RT-qPCR and Western blot to detect changes in CTLA4 expression in T cells with MAF knockdown after hrCHI3L1 treatment. J, Representative results (left) and quantitative statistical chart (right) of changes in the proportion of CTLA4^+^ T cells with MAF knockdown after hrCHI3L1 treatment as detected by flow cytometry. ^ns^ *p* > 0.05, * *p* < 0.05, ** *p* < 0.01, *** *p* < 0.001. Cell experiments were repeated three times.

**Additional file 6: Figure S6.** The expression distribution of CD80 and CD86 in scRNA-seq. A & B, The expression distribution of CD80 and CD86 in the scRNA-seq, respectively.

**Additional file 7: Figure S7.** DEGs in CTLA4^+^ T cells and G.O. enrichment analysis. A Volcano map of DEGs in CTLA4^+^ T cells. Red represents upregulated genes, and green represents downregulated genes. B & C, G.O. enrichment analysis results of upregulated genes (B) and downregulated genes (C) in CTLA4^+^ T cells.

**Additional file 8: Table S1.** shRNA sequences. **Table S2.** Manufacturer information of primary antibodies. **Table S3.** Primer sequences for RT-qPCR.

## Data Availability

The data supporting this study's findings are available on request from the corresponding author.

## Abbreviations

TNBC: Triple-negative breast cancer
TN-BCSCs: Triple-negative breast cancer stem cells
CTLA4: Cytotoxic T lymphocyte antigen 4
PCA: Principal component analysis
DEGs: Differentially expressed genes
ELISA: Enzyme-linked immunosorbent assay
