# Supplementary material for: Cancer stem cell-derived CHI3L1 activates the MAF/CTLA4 signaling pathway to promote immune escape in triple-negative breast cancer
Source: J Transl Med. 2023 Oct 14;21:721. doi: 10.1186/s12967-023-04532-6 (PMC10576881; doi:10.1186/s12967-023-04532-6)
Supplement: Supplementary file 8 — Additional file 8: Table S1. shRNA sequences. Table S2. Manufacturer information of primary antibodies. Table S3. Primer sequences for RT-qPCR. [file 12967_2023_4532_MOESM8_ESM.docx]

**Table S1** shRNA sequences

| shRNA | Sequences |
| --- | --- |
| sh-NC | 5’-CCTAAGGTTAAGTCGCCCTCG-3’ |
| sh-MAF-1 | 5’-CAAGGAGAAATACGAGAAGTT-3’ |
| sh-MAF-2 | 5’-ACAAGGAGAAATACGAGAAGT-3’ |

Note: sh-, short hairpin RNA-; NC, negative control.

**Table S2** Manufacturer information of primary antibodies

| Antibody | Manufacturer | Cat. | Dilution ratio |
| --- | --- | --- | --- |
| CHI3L1 (Human/Mouse) | Abcam | ab180569 | 1:500 |
| CTLA4 (Human/Mouse) | Abcam | ab237712 | 1:1000 |
| MAF (Human/Mouse) | Abcam | ab243901 | 1:1000 |
| CD44 (Human/Mouse) | Abcam | ab243894 | 1:1000 |
| CD24 (Human) | Abcam | ab179821 | 1:1000 |
| β-actin (Human/Mouse) | Abcam | ab8226 | 1:1000 |

Note: CHI3L1, chitinase 3 like 1; CTLA4, cytotoxic T-lymphocyte associated protein 4.

**Table S3** Primer sequences for RT-qPCR

| Gene | Sequences |
| --- | --- |
| CHI3L1 (Human) | Forward: 5’-AAGCAACGATCACATCGACAC-3’ |
|  | Reverse: 5’-TCAGGTTGGGGTTCCTGTTCT-3’ |
| CTLA4 (Human) | Forward: 5’-CATGATGGGGAATGAGTTGACC-3’ |
|  | Reverse: 5’-TCAGTCCTTGGATAGTGAGGTTC-3’ |
| MAF (Human) | Forward: 5’-CTGGCAATGAGCAACTCCGA-3’ |
|  | Reverse: 5’-AGCCGGTCATCCAGTAGTAGT-3’ |
| S100A4 (Human) | Forward: 5’-GATGAGCAACTTGGACAGCAA-3’ |
|  | Reverse: 5’-CTGGGCTGCTTATCTGGGAAG-3’ |
| CD44 (Human) | Forward: 5’-CTGCCGCTTTGCAGGTGTA-3’ |
|  | Reverse: 5’-CATTGTGGGCAAGGTGCTATT-3’ |
| CD24 (Human) | Forward: 5’-CTCCTACCCACGCAGATTTATTC-3’ |
|  | Reverse: 5’-AGAGTGAGACCACGAAGAGAC-3’ |
| β-actin (Human) | Forward: 5’-GAGAAAATCTGGCACCACACC-3’ |
|  | Reverse: 5’-GGATAGCACAGCCTGGATAGCAA-3’ |

Note: RT-qPCR, reverse transcription quantitative real-time polymerase chain reaction; CHI3L1, chitinase 3 like 1; CTLA4, cytotoxic T-lymphocyte associated protein 4; S100A4, S100 calcium binding protein A4.
